# Single-Visit Pulp Revascularization of a Nonvital Immature Permanent Tooth Using Biodentine 

**DOI:** 10.7508/iej.2016.03.020

**Published:** 2016-05-01

**Authors:** Mohammad Mhd Nader Aldakak, Ismail Davut Capar, Mohammad Salem Rekab, Souad Abboud

**Affiliations:** a Damascus University, Syrian Arab Republic;; b Izmir Katip Çelebi University, Izmir, Turkey

**Keywords:** Biodentine, Immature Apex, Pulp Revascularization

## Abstract

An 11-year-old female patient was referred with a chief complaint of pain in the right mandibular second premolar. Clinical and radiographic examinations showed secondary caries under an old composite restoration, a negative response to a pulp test and an immature root with an open apex. After root canal cleaning and shaping, bleeding was invoked in the canal up to 2 mm short of the cemento-enamel junction (CEJ). After 10 min to allow clotting at this level, a plug of Biodentine was placed over the blood clot and the tooth was temporized with glass ionomer cement. At the next visit, the tooth was free of symptoms and a permanent filling was placed. Clinical and radiographic examinations during a two-year follow-up showed complete root maturation and intact supporting soft tissues without sinus tract, pain or swelling.

**Conclusion:**

The use of Biodentine in a single-visit apexification protocol to treat an immature permanent tooth with necrotic pulp can create a suitable environment for revascularization, resulting in the completion of root maturation.

## Introduction

Presence of an immature apex in a tooth with pulpal injury presents a significant challenge, as routine root canal procedures cannot be performed ideally when the apex is not closed [1]. Thus, the results of treatment are unpredictable. Depending on the vitality of the affected pulp, there are two possible approaches: apexogenesis, which is used when the pulp is vital and apexification, applied for necrotic pulps [2]. In immature nonvital teeth, the root canal walls diverge apically, which make the preparation of an apical stop impossible. In such cases, the aim of treatment is to induce the formation of an apical buttress of hard tissue and avoid overfilling of the canal obturating materials (*aka *apexification) [3]. 

The proper approach in this treatment modality is known as apexification, the goal of which is to establish an apical stop [4]. Many materials, such as non-setting calcium hydroxide (CH), have been reported to successfully stimulate apexification. The main disadvantage of this technique is the increased possibility of cervical fracture [5], as well as the frequent number of clinical visits required to complete this procedure. 

The use of tricalcium phosphate as an apical barrier in single-visit apexification procedure was reported in 1979; the material was packed into the apical 2 mm of the canal, against which gutta-percha was compacted. The radiographic assessment confirmed successful apexification comparable to the results achieved with CH [6]. 

With regards to one-step apexification, placement of mineral trioxide aggregate (MTA) apical plug has a number of advantages, such as decreasing the number of appointments and reducing the clinical time. However, this method does not facilitate further root development [7].

In regenerative procedures, the treatment goal is to induce biological replacement of lost dental tissue(s). Many of these procedures have emerged from the growing field of tissue engineering [8]. Over the last several decades, the scope and clinical application of regenerative dental procedures have continuously advanced to include guided tissue regeneration (GTR), guided bone regeneration (GBR) and distraction osteogenesis (DO) [9]. Regenerative endodontics, such as pulp revascularization, has been defined as biologically based procedures designed to replace damaged structures, such as dentin, root structures and cells of the pulp-dentin complex [10]. Pulp revascularization has been widely performed for the treatment of immature permanent teeth with necrotic pulps and established apical periodontitis. Successful cases exhibited thickening of the canal walls, closure of root apices and continued root development [11]. Today, the regeneration of immature permanent teeth with necrotic pulps can be achieved using different types of biomaterials which may require multiple or single visits [12].

There are many different multiple-step revascularization protocols, with different kinds of intracanal medicaments such as CH. A previous study demonstrated that root fracture was the main reason for tooth loss after apexification with CH in a large number of cases [13]. A CH dressing was also shown to be less effective than antibiotic paste formulations against some intracanal bacteria [14].

Another dressing, triple antibiotic paste (TAP) was suggested to disinfect the canal. A study investigated the use of TAP as intracanal medicament. In that study, 51% of revascularization cases were successfully treated with TAP (a 1:1:1 mixture of ciprofloxacin/metronidazole/minocycline) [15]. Other studies showed that TAP was very effective against endodontic microorganisms [16]. However, TAP also caries many drawbacks. For example, researches have demonstrated that more than 80% of the paste could not be removed from the tooth and that it penetrated through the dentinal tubules rather than remaining in the canal lumen [17]. Another study noted that it was difficult to completely remove the paste from the root canals [18]. Furthermore, the combination of TAP and CH is not approved by the US Food and Drug Administration (FDA) and has the potential to induce staining of the crown [17]. In addition, TAP was shown to be toxic to stem cells, with one study reporting that higher TAP concentrations had a detrimental effect on stem cells of apical papilla [19].

Shin *et al.* [12] previously described the first and only revascularization case completed in one visit. However, they used NaOCl irrigation to disinfect the canal, followed by MTA to seal the canal orifices. Recent studies have demonstrated that the performance of Biodentine (Septodont, St. Maur-des-Fosses, France) was equal or superior to that of MTA. However, to the best of our knowledge, there are no reports on the use of Biodentine in a single-visit revascularization procedure. This case report represents the application of Biodentine in a single-visit protocol for revascularization of an immature mandibular premolar.

## Case Report

**Figure 1:**
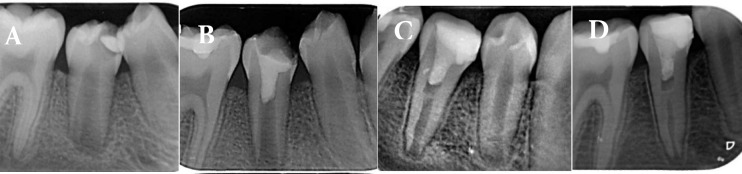
Preoperative radiography, B) Six-month follow-up after revascularization, C) Twelve-month follow-up, D) Two-year follow-up

An 11-year-old female patient was referred to the clinic of Endodontics and Operative Dentistry at Damascus University, Dental School with a chief complaint of pain in the right mandibular second premolar. Vitality, percussion and palpation tests of the mandibular second premolar and adjacent teeth revealed its negative response and positive responses of adjacent teeth to pulp vitality tests. An evaluation radiography revealed the presence of secondary caries under an old composite restoration on the immature second premolar. All the tests were done using Endo-Frost (Coltène-Whaledent, Langenau, Germany) (Figure 1A). The final diagnosis was pulp necrosis of the immature tooth.

Local anesthesia was administrated by infiltration of mepivacaine (Mepivacaine HCL 3%, Alexandria, Egypt). The tooth was isolated using a rubber dam and the access cavity was prepared. The canal was initially irrigated with a 5.25% NaOCl solution. The working length was established radiographically and confirmed with an apex locater (J. Morita USA, Inc., Irvine, CA, USA). The canal was irrigated with the needle inserted 2 mm shorter than the working length, using 17% ethylenediaminetetraacetic acid (EDTA, Dia-Prep Plus, Diadent Group International Inc., Chongju, Korea) for 1 min to remove the smear layer, without damaging the stem cells present in the periapical area [20]. The canal was then irrigated with saline, followed by irrigation with 5.25% NaOCl for 3 min, saline and then 2% chlorhexidine for 5 min. Finally, the canal was irrigated with saline and dried using sterilized paper points. A #20 K-file (Dentsply Maillefer, Ballaigues, Switzerland) was then used to irritate the apical tissues gently and invoke bleeding in the canal. The bleeding was stopped 2 mm below the CEJ level and left for 10 min to allow clottting.

A mixture of Biodentine (BD, Septodont, Saint Maur des Fosses, France) was applied according to the manufacturer’s instructions and placed over the blood clot using endodontic pluggers. After 15 min, a light-cure glass ionomer restoration (GC Fuji II LC, GC Corp, Tokyo, Japan) was placed, and the patient was scheduled for follow-up (Figure 1B). Six months later, the teeth were double-sealed with permanent filling materials (3M ESPE, FiltekTM Z250, Universal Restorative, USA). The patient was recalled after 6, 12, and 24 months for clinical and radiographic examinations, which showed complete root maturation, with intact supporting soft tissues, without a sinus tract, pain, or swelling (Figures 1C and 1D).

## Discussion

According to the approach presented by Nygaard-Ostby [21], which is based on the well-known role of blood clot formation in wound healing, pulp regeneration involves laceration of the periapical tissues. Their study reported histologic and clinical findings on 17 teeth that were extracted after 3 years of follow-up, the growth of periodontal ligament and periapical bone were incomplete, and the authors reported evidences of variable resorption of the dentinal walls, along with the deposition of cementum. Thus no newly formed dentin was observed [21].

The growth and differentiation of mesenchymal stem cells from periapical cells, depend on blood clot formation that serves as a scaffold, enabling the migration of stem cells into the root canal where they then stimulate the formation of new tissues in the canal space [22]; however, the ingrowth of periodontal tissues that reach the coronal pulp chamber it is very likely [23].

It was reported that teeth with an apical foramen larger than 1.1 mm, are potentially suitable candidates for revascularization after replantation [24]. An earlier study on partially necrotic young teeth reported a strong relationship between pulp and periapical tissues, although the coronal part was infected, the apical part of the pulp tissue retained its vitality [25]. Another study reported that vital pulp cells in the apical part had the ability to create new pulpal tissues in the coronal pulp space, provided that the coronal part was disinfected [26]. Therefore, as noted elsewhere, obturating the root canal with filling materials or medicaments eliminate the chances of revascularization and is counterproductive [27].

There are many probable means by which revascularization of pulpal tissue can occur. *First*, revascularization of vital pulpal cells can occur at the apical end of the root canal when the dental papilla is located apical to the developing pulp during root and pulp development [26]. Dental papilla is histologically different from the pulp and the apical papilla is less vascular and cellular; these two tissues are separated by a cell-rich zone [28]. *Second*, stem cells from the apical papilla or bone marrow can trigger root development [29]. In addition, multipotent dental pulp stem cells can play a role in continued root development [30]. These cells are present in permanent teeth and might be plentiful in immature teeth. *Third*, revascularization of pulpal tissues may also be induced by stem cells present in the periodontal ligament [31]. *Finally*, the blood clot itself may aid the revascularization of pulp tissue because it includes many growth factors, which have a crucial role in regeneration [32].

A previous study described three components that contributed to successful revascularization [33]. These were stem cells that are capable of inducing the formation of hard tissues; signaling molecules for cellular stimulation, proliferation and differentiation; and a three-dimensional physical scaffold that can support cell growth and differentiation [33]. To facilitate the revascularization of immature teeth with apical periodontitis, the following three steps are required: The canal must be disinfected; an intracanal matrix should be put in place for tissue in-growth and a tight coronal seal should be applied to prevent bacterial (re)entry [34]. 

Completing revascularization in one visit has many advantages. First, it reduces the chance of additional bacterial contamination of the space. Second, a single revascularization protocol may overcome the problem of poor patient compliance with follow-up visits and reduce the number of appointments needed. Third, it can help to combat potential injury of the tooth [12]. In the present case, Biodentine was used as a plug in the canal orifice of the canal under the CEJ [35] rather than MTA because it was shown that Biodentine did not change the color of the tooth, whereas MTA caused discoloration [36]. With regard to the biocompatibility of Biodentine, a previous study demonstrated that it was comparable to that of MTA [37]. The similar biocompatibility is primarily due to the composition of the materials (*i.e.*, mainly tricalcium silicate).

A study showed that the only difference in the pulpal response to Biodentine and MTA was in the thickness of the mineralized tissue bridge, which was greater with Biodentine [38]. The result of that study may be explained by the difference in the velocity of the chemical reaction during the setting of the materials. Although both materials produce the same chemical compounds, this reaction may be faster in Biodentine, which has a shorter setting time. Based on the aforementioned research, it was decided to use Biodentine instead of MTA in the present case.

## Conclusion

It appears that using Biodentine in a single-visit revascularization protocol can aid in root development and continued apical closure.

## References

[1] Tabrizizade M, Asadi Y, Sooratgar A, Moradi S, Sooratgar H, Ayatollahi F (2014). Sealing ability of mineral trioxide aggregate and calcium-enriched mixture cement as apical barriers with different obturation techniques. Iran Endod J.

[2] Capurro M, Zmener O (1999). Delayed apical healing after apexification treatment of non-vital immature tooth: a case report. Endod Dent Traumatol.

[3] Silva RV, Silveira FF, Nunes E (2015). Apexification in non-vital teeth with immature roots: report of two cases. Iran Endod J.

[4] Beer R, Ballesio I, Kielbassa A (2005). Pocket Atlas of Endodontics.

[5] Andreasen JO, Farik B, Munksgaard EC (2002). Long-term calcium hydroxide as a root canal dressing may increase risk of root fracture. Dent Traumatol.

[6] Schumacher JW, Rutledge RE (1993). An alternative to apexification. J Endod.

[7] Felippe WT, Felippe MC, Rocha MJ (2006). The effect of mineral trioxide aggregate on the apexification and periapical healing of teeth with incomplete root formation. Int Endod J.

[8] Langer R, Vacanti JP (1993). Tissue engineering. Science.

[9] Oh SL, Fouad AF, Park SH (2009). Treatment strategy for guided tissue regeneration in combined endodontic-periodontal lesions: case report and review. J Endod.

[10] Murray PE, Garcia-Godoy F, Hargreaves KM (2007). Regenerative endodontics: a review of current status and a call for action. J Endod.

[11] Wang Y, Zhu X, Zhang C (2015). Pulp Revascularization on Permanent Teeth with Open Apices in a Middle-aged Patient. J Endod.

[12] Shin SY, Albert JS, Mortman RE (2009). One step pulp revascularization treatment of an immature permanent tooth with chronic apical abscess: a case report. Int Endod J.

[13] Cvek M (1992). Prognosis of luxated non-vital maxillary incisors treated with calcium hydroxide and filled with gutta-percha. A retrospective clinical study. Endod Dent Traumatol.

[14] Sabrah AH, Yassen GH, Gregory RL (2013). Effectiveness of antibiotic medicaments against biofilm formation of Enterococcus faecalis and Porphyromonas gingivalis. J Endod.

[15] Diogenes AR, Ruparel NB, Teixeira FB, Hargreaves KM (2014). Translational science in disinfection for regenerative endodontics. J Endod.

[16] Sato T, Hoshino E, Uematsu H, Noda T (1993). In vitro antimicrobial susceptibility to combinations of drugs on bacteria from carious and endodontic lesions of human deciduous teeth. Oral Microbiol Immunol.

[17] Berkhoff JA, Chen PB, Teixeira FB, Diogenes A (2014). Evaluation of triple antibiotic paste removal by different irrigation procedures. J Endod.

[18] Arslan H, Akcay M, Capar ID, Ertas H, Ok E, Uysal B (2014). Efficacy of needle irrigation, EndoActivator, and photon-initiated photoacoustic streaming technique on removal of double and triple antibiotic pastes. J Endod.

[19] Ruparel NB, de Almeida JF, Henry MA, Diogenes A (2013). Characterization of a stem cell of apical papilla cell line: effect of passage on cellular phenotype. J Endod.

[20] Trevino EG, Patwardhan AN, Henry MA, Perry G, Dybdal-Hargreaves N, Hargreaves KM, Diogenes A (2011). Effect of irrigants on the survival of human stem cells of the apical papilla in a platelet-rich plasma scaffold in human root tips. J Endod.

[21] Nygaard-Ostby B, Hjortdal O (1971). Tissue formation in the root canal following pulp removal. Scand J Dent Res.

[22] Thibodeau B, Teixeira F, Yamauchi M, Caplan DJ, Trope M (2007). Pulp revascularization of immature dog teeth with apical periodontitis. J Endod.

[23] Nevins A, Wrobel W, Valachovic R, Finkelstein F (1977). Hard tissue induction into pulpless open-apex teeth using collagen-calcium phosphate gel. J Endod.

[24] Kling M, Cvek M, Mejare I (1986). Rate and predictability of pulp revascularization in therapeutically reimplanted permanent incisors. Endod Dent Traumatol.

[25] Iwaya SI, Ikawa M, Kubota M (2001). Revascularization of an immature permanent tooth with apical periodontitis and sinus tract. Dent Traumatol.

[26] Banchs F, Trope M (2004). Revascularization of immature permanent teeth with apical periodontitis: new treatment protocol?. J Endod.

[27] Akgun OM, Altun C, Guven G (2009). Use of triple antibiotic paste as a disinfectant for a traumatized immature tooth with a periapical lesion: a case report. Oral Surg Oral Med Oral Pathol Oral Radiol Endod.

[28] Sonoyama W, Liu Y, Yamaza T, Tuan RS, Wang S, Shi S, Huang GT (2008). Characterization of the apical papilla and its residing stem cells from human immature permanent teeth: a pilot study. J Endod.

[29] Gronthos S, Mankani M, Brahim J, Robey PG, Shi S (2000). Postnatal human dental pulp stem cells (DPSCs) in vitro and in vivo. Proc Natl Acad Sci U S A.

[30] Gronthos S, Brahim J, Li W, Fisher LW, Cherman N, Boyde A, DenBesten P, Robey PG, Shi S (2002). Stem cell properties of human dental pulp stem cells. J Dent Res.

[31] Lieberman J, Trowbridge H (1983). Apical closure of nonvital permanent incisor teeth where no treatment was performed: case report. J Endod.

[32] Shah N, Logani A, Bhaskar U, Aggarwal V (2008). Efficacy of revascularization to induce apexification/apexogensis in infected, nonvital, immature teeth: a pilot clinical study. J Endod.

[33] Hargreaves KM, Giesler T, Henry M, Wang Y (2008). Regeneration potential of the young permanent tooth: what does the future hold?. J Endod.

[34] Windley W, 3rd, Teixeira F, Levin L, Sigurdsson A, Trope M (2005). Disinfection of immature teeth with a triple antibiotic paste. J Endod.

[35] Asgary S, Fazlyab M (2015). A Successful Endodontic Outcome with Non-Obturated Canals. Iran Endod J.

[36] Camilleri J (2015). Staining Potential of Neo MTA Plus, MTA Plus, and Biodentine Used for Pulpotomy Procedures. J Endod.

[37] Attik GN, Villat C, Hallay F, Pradelle-Plasse N, Bonnet H, Moreau K, Colon P, Grosgogeat B (2014). In vitro biocompatibility of a dentine substitute cement on human MG63 osteoblasts cells: Biodentine versus MTA((R)). Int Endod J.

[38] De Rossi A, Silva LA, Gaton-Hernandez P, Sousa-Neto MD, Nelson-Filho P, Silva RA, de Queiroz AM (2014). Comparison of pulpal responses to pulpotomy and pulp capping with biodentine and mineral trioxide aggregate in dogs. J Endod.

